# Successful endoscopic submucosal dissection of a duodenal neuroendocrine tumor close to the major papilla with traction from rubber band and clips

**DOI:** 10.1055/a-2558-5086

**Published:** 2025-05-12

**Authors:** Xiwei Ding, Guifang Xu, Shanshan Shen, Lei Wang

**Affiliations:** 1 Department of Gastroenterology, Nanjing Drum Tower Hospital, Affiliated Hospital of Medical School, Nanjing University, Nanjing, China

Endoscopic submucosal dissection (ESD) has been shown to be effective for treatment of small nonampullary duodenal neuroendocrine tumors (NETs) 1 2 . However, ESD may be challenging when a tumor is close to the duodenal major papilla. Herein, we report use of ESD with traction to successfully remove a duodenal NET located just below the papilla without adverse events (AEs) in a 53-year-old woman ( Video 1 ).

Endoscopic submucosal dissection of a duodenal neuroendocrine tumor close to the major papilla with rubber band traction.Video 1

## Case report

**Fig. 1:**
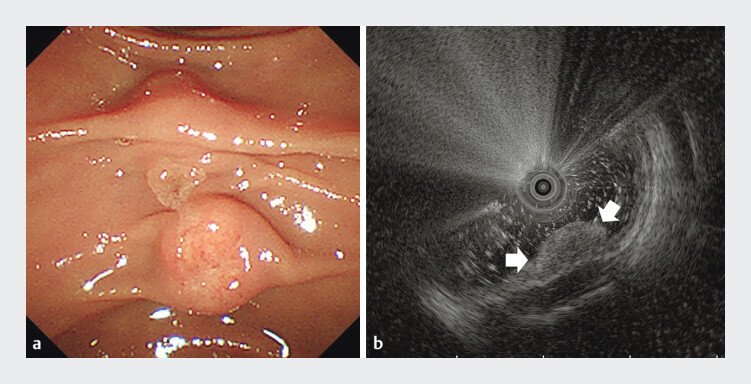
Duodenoscopy showed a subepithelial tumor-like lesion in the duodenum close to the major papilla. **b** Endoscopic ultrasonography revealed a low echogenic mass arising from submucosal layer.

**Fig. 2:**
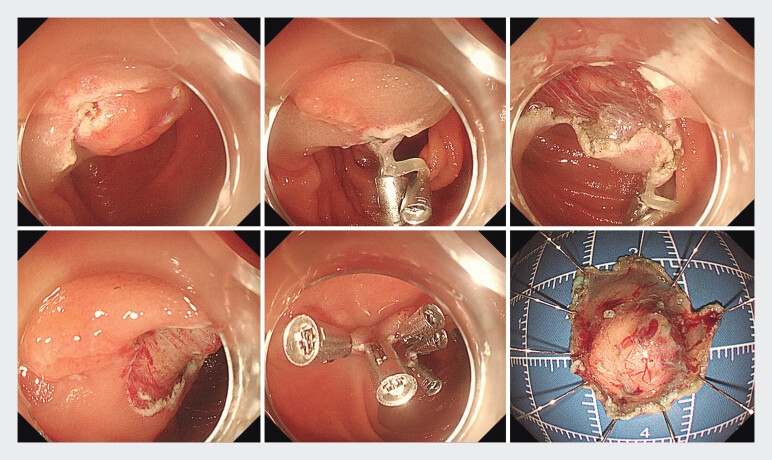
**a** Incision of the oral side. **b** A rubber band and double clips were used for traction. **c** Endoscopic submucosal dissection was performed under traction. **d** Resected area. **e** The wound was closed with clips. **f** Resected specimen.

**Fig. 3:**
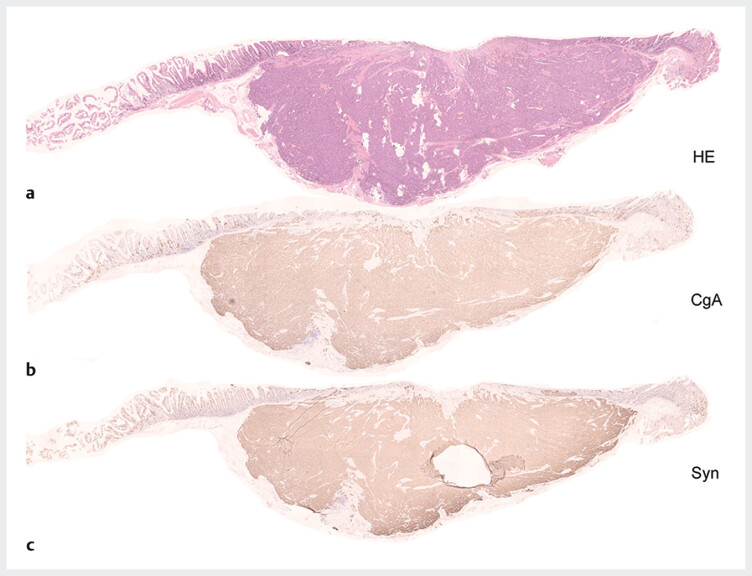
**a** Hematoxylin and eosin staining of the tumor showed neuroendocrine tumor with negative margins. **b** The tumor was positive for chromogranin A. **c** . The tumor was positive for synaptophysin.

The patient underwent esophagogastroduodenoscopy due to epigastric discomfort. A subepithelial tumor-like mass was found in the duodenum. Biopsy of the lesion confirmed the NET. Duodenoscopy showed the tumor located just below the papilla ( Fig. 1 **a** ). Endoscopic ultrasonography revealed a 8.6 mm×4.1 mm low echogenic mass arising from the submucosal layer ( Fig. 1 **b** ). ESD was performed to ensure R0 resection. After incision of the oral side, traction was performed with a rubber band and double clips ( Fig. 2 **a–c** ). Then, dual knife and IT nano knife were used to dissect the submucosal layer successfully without damage to the major papilla under the traction. After removing the lesion, the wound was closed with endoscopic clips ( Fig. 2 **d, Fig. 2 e, Fig. 2 f** ). Procedure time was 26 minutes. Starting 24 hours after the procedure, the patient was given a liquid diet. The patient was discharged 4 days after the ESD without any AEs. Pathology showed NET G1 with negative margins ( Fig. 3 **a** ). The tumor was positive for chromogranin A and synaptophysin on immunohistochemistry ( Fig. 3 **b, Fig. 3 c** ). There was no vascular invasion. The tumor size was 8 mm×6 mm.

## Conclusions

ESD with rubber band traction may be effective and safe for removing small duodenal NETs close to major papilla.
